# Continuation of burosumab during pregnancy in a patient with X-linked hypophosphatemia

**DOI:** 10.1210/jcemcr/luag233

**Published:** 2026-08-24

**Authors:** Atsushi Suzuki, Yoshinori Moriyama, Haruo Mizuno, Haruki Nishizawa

**Affiliations:** Department of Endocrinology, Diabetes and Metabolism, Fujita Health University School of Medicine, Toyoake, Aichi 470-1192, Japan; Department of Obstetrics and Gynecology, Fujita Health University School of Medicine, Toyoake, Aichi 470-1192, Japan; Department of Pediatrics, Fujita Health University School of Medicine, Toyoake, Aichi 470-1192, Japan; Department of Obstetrics and Gynecology, Fujita Health University School of Medicine, Toyoake, Aichi 470-1192, Japan

**Keywords:** X-linked hypophosphatemia, burosumab, pregnancy, FGF23, PHEX

## Abstract

Burosumab is a well-established treatment for X-linked hypophosphatemia (XLH), but safety data during pregnancy are limited. We present the case of a woman in her 30s with XLH who continued to take burosumab during pregnancy to prevent symptomatic relapse. She gave birth to a male infant via cesarean section at 37 weeks without obstetric complications. The neonate exhibited a markedly elevated intact fibroblast growth factor 23 level {48 700 pg/mL (SI: 1933 pmol/L) (reference range, 19.9-52.9 pg/mL [SI: 0.79-2.10 pmol/L])} shortly after birth. This level declined rapidly to 151 pg/mL (SI: 4.7 pmol/L) by age 7 months but remained above the normal range. Genetic testing revealed a *phosphate-regulating endopeptidase, X-linked* gene mutation in both mother and child. The infant was initially managed with conventional therapy before transitioning to burosumab at 8 months of age. At the 1-year follow-up, the child demonstrated no apparent developmental delay or adverse skeletal events. This case presents real-world evidence of continued burosumab exposure during pregnancy in a woman with XLH. Although no apparent short-term maternal or fetal adverse events were observed, the safety of burosumab use during pregnancy is uncertain, and more clinical data, particularly with long-term follow-up, are needed.

## Introduction

X-linked hypophosphatemia (XLH) is a rare, lifelong disorder caused by loss-of-function variants in the *phosphate-regulating endopeptidase, X-linked* (*PHEX*) gene, which results in excessive fibroblast growth factor 23 (FGF23) activity. Chronic hypophosphatemia in adults causes osteomalacia, leading to pseudofractures, osteoarthritis, enthesopathy, and severe musculoskeletal pain and stiffness [1]. Burosumab, a fully human IgG1 monoclonal antibody that targets FGF23, has significantly improved phosphate metabolism, fracture healing, and physical function in adults with XLH [2]. Long-term studies have shown these benefits persist [3]. In contrast, conventional therapy is associated with complications, such as nephrocalcinosis and secondary/tertiary hyperparathyroidism. A recent large cohort study in Asian patients found that hyperparathyroidism and renal dysfunction are significant comorbidities in those receiving conventional therapy [4]. However, treating XLH during pregnancy remains challenging. The most recent international clinical practice guidelines recommend conventional therapy during pregnancy, recognizing that data on burosumab safety in this context are insufficient [5, 6]. However, discontinuing burosumab may cause rapid recurrence of debilitating symptoms and compromise maternal well-being. Here, we present a case of a woman with XLH who received burosumab throughout pregnancy and discuss the clinical implications, particularly regarding the interpretation of markedly elevated FGF23 levels observed in the neonate.

## Case presentation

**Figure 1:**
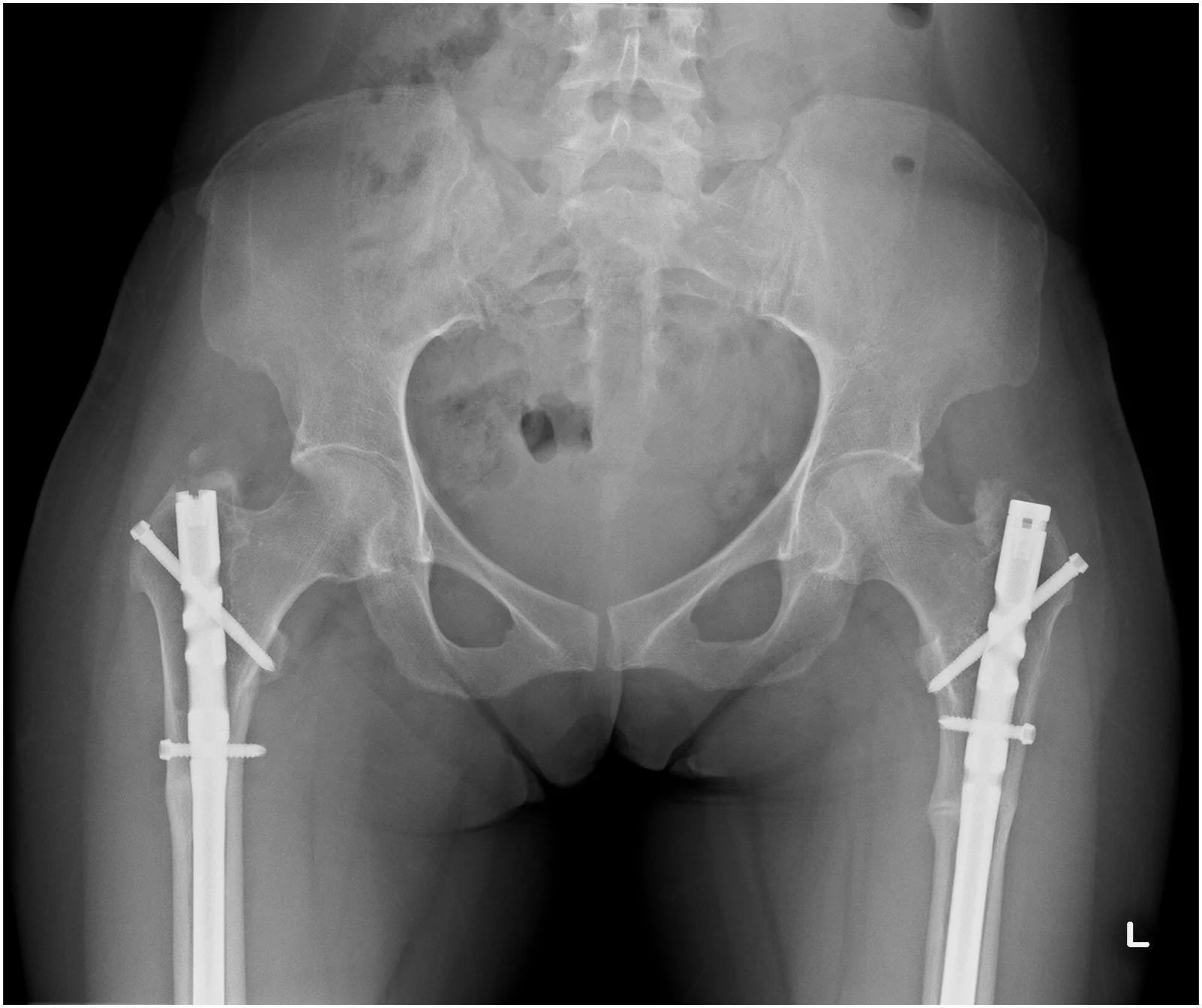
Radiographic finding of the mother. Anteroposterior radiograph of the mother's hips at age 32, with intramedullary nails inserted for femoral fractures, demonstrating the severity of her bone disease prior to burosumab treatment.

The patient was a woman in her 30s diagnosed with XLH at 3 years of age. Her height was 148.1 cm, and her weight was 46.1 kg. Despite receiving conventional therapy as a child and corrective surgery at the age of 15, she experienced recurrent fractures and persistent bone pain as an adult. At the age of 31, she suffered femoral fractures requiring intramedullary fixation (Fig. 1). She had no history of treatment interruption and maintained excellent medication adherence throughout the course. She presented at the age of 32 with severe bone pain and needed a cane to walk. Her biochemical profile showed serum phosphate at the lower limit of the reference range (serum phosphate of 2.7 mg/dL [SI: 0.87 mmol/L]) (reference range, 2.7-4.6 mg/dL [SI: 0.87-1.49 mmol/L]) along with elevated intact FGF23 (78.1 pg/mL [SI: 3.1 pmol/L]) (reference range, 19.9-52.9 pg/mL [SI: 0.79-2.10 pmol/L]) and renal phosphate wasting, with tubular reabsorption of phosphate (TmP/GFR) of 1.70 mg/dL (SI: 0.55 mmol/L) (reference range, 2.6-4.4 mg/dL [SI: 0.84-1.41 mmol/L]), despite treatment with alfacalcidol (0.75 mcg/day) and oral phosphate (1500 mg/day).

## Diagnostic assessment

The diagnosis was made clinically based on rickets history and biochemical results. Prior to pregnancy, the patient received genetic counseling as part of her marriage, followed by genetic testing.

Since 16 genes cause hereditary hypophosphatemic rickets, short-read next-generation sequencing was performed as a panel test. This revealed a disruption of sequencing reads at chrX:22,099,026 within the *PHEX* gene, raising suspicion of a heterozygous deletion/insertion (indel) variant originating at this locus; however, a definitive molecular diagnosis could not be established using this method. The clinical response to burosumab (50 mg subcutaneously every 4 weeks) was robust; within 7 months, her bone pain subsided, and she no longer needed a cane. Serum phosphate was normalized to 3.2 mg/dL (SI: 1.03 mmol/L) with an improvement in TmP/GFR (2.80 mg/dL [SI: 0.90 mmol/L]). Prior to conception, the patient received multidisciplinary counseling regarding pregnancy and burosumab treatment. She was informed that there were limited safety data on burosumab use during pregnancy and that current guidelines generally recommend conventional therapy during pregnancy. The discussion also included the possibility of transplacental transfer of burosumab as an IgG1 monoclonal antibody as well as the 50% risk of transmitting the *PHEX* variant to the offspring, with the possibility that fetal exposure to burosumab could have different consequences depending on whether the fetus inherited XLH. After understanding these uncertainties, the patient expressed a strong desire to continue burosumab therapy if pregnancy occurred. The pregnancy was confirmed at 34 years and 9 months of age, 20 months after starting burosumab. The patient expressed strong concern about symptom recurrence and potential loss of mobility if treatment was discontinued and expressed a strong preference to continue receiving burosumab.

## Treatment

After extensive discussions of limited burosumab safety data during pregnancy, the potential for transplacental drug transfer, and the uncertain fetal effects depending on the inheritance of the *PHEX* variant, a shared decision was made to continue burosumab at the same dose (50 mg every 4 weeks) throughout pregnancy with close maternal and fetal monitoring; conventional therapy was not reintroduced.

## Outcome and follow-up

The pregnancy was relatively uneventful, except for threatened preterm labor at 33 weeks, which was managed conservatively. Serial ultrasonography revealed normal fetal growth with no skeletal anomalies. At 35 years and 4 months of age, because of the patient's history of recurrent fractures associated with XLH and concerns about muscle weakness resulting in prolonged or difficult labor, an elective cesarean section was scheduled at 37 weeks and 0 days to reduce mechanical stress on the maternal skeleton. A male infant weighing 2325 g was delivered with Apgar scores of 8 and 8 at 1 and 5 minutes, respectively. The placenta exhibited mild signs of villous immaturity but no calcification.

**Figure 2:**
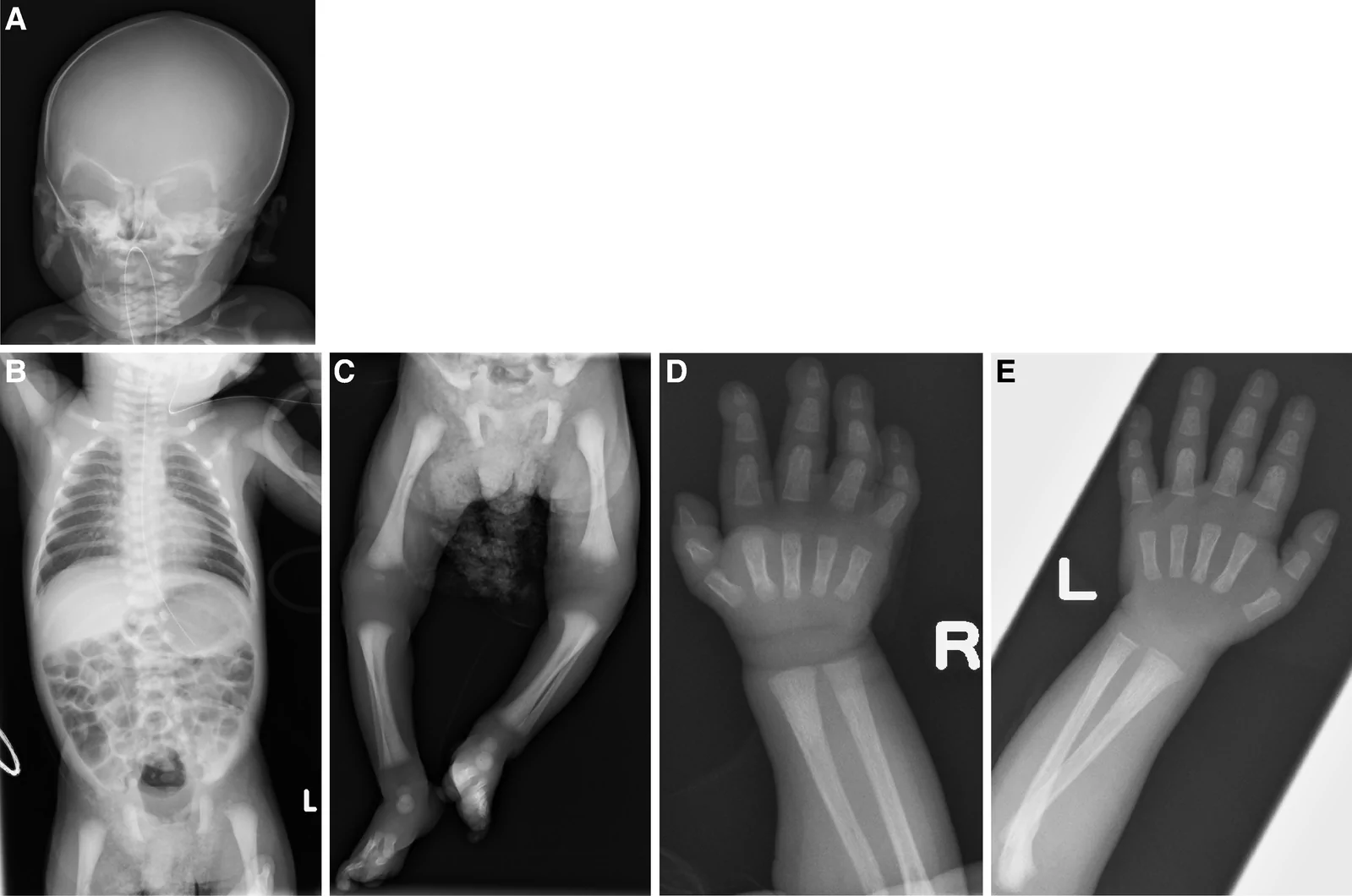
Radiographic findings of the infant. (A-C) Radiograph of the neonate on day 2. Despite extremely high serum FGF23 levels, there is no evidence of rickets or skeletal dysplasia. (D-E) Radiograph of the infant's hands at 1 month of age, revealing normal mineralization.

**Figure 3:**
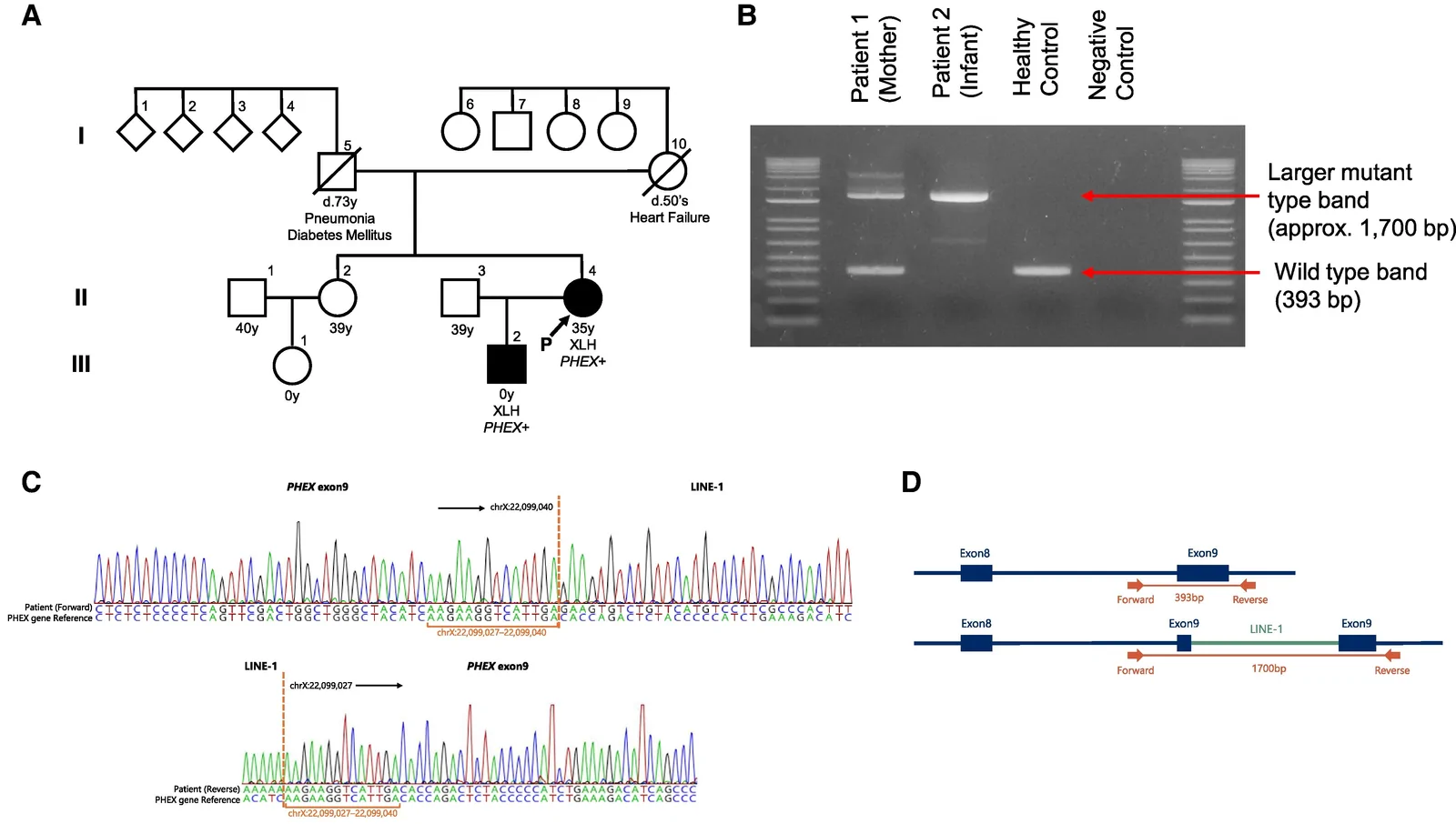
Genetic analysis of the *PHEX* gene. (A) Pedigree of the family at the time of birth of the affected infant (III-2). Filled symbols show individuals affected by XLH. The arrow denotes the proband (II-4). “*PHEX*+” indicates individuals in whom the identified *PHEX* insertion variant was confirmed by molecular testing. Deceased individuals are represented by a diagonal slash. Individual numbers are shown in the upper right corner of each symbol, and generation numbers are indicated on the left side of the pedigree. (B) PCR analysis of the *PHEX* gene. Gel electrophoresis reveals a wild-type band (393 bp) and a larger mutant band (approximately 1700 bp) in both the mother and infant, indicating a large insertion. (C) Nucleotide sequence of the *PHEX* insertion site determined using Sanger sequencing. PCR products spanning the insertion breakpoint were subjected to Sanger sequencing. A LINE-1-derived sequence was identified immediately downstream of exon 9 of the *PHEX* gene (chrX:22,099,040). A 14-base pair (bp) target site duplication (chrX:22,099,027-22,099,040) flanking the inserted sequence was observed. (D) Schematic representation of the *PHEX* gene mutation identified by long-read sequencing, showing a LINE-1 retrotransposon insertion near exon 9. Approx., approximately.

The neonate was admitted to the neonatal intensive care unit with apnea but quickly stabilized. On day 2 after birth, laboratory evaluation revealed an extremely high intact FGF23 level of 48 700 pg/mL (SI: 1933 pmol/L), along with serum phosphate of 4.2 mg/dL (SI: 1.36 mmol/L) (reference range for newborn, 5.0-7.7 mg/dL [SI: 1.61-2.49 mmol/L]) and 1,25-dihydroxyvitamin D of 57.8 pg/mL (SI: 139 pmol/L) (reference range, 20.0-70.0 pg/mL [SI: 48.1-168.3 pmol/L]) (Table 1). Skeletal radiographs revealed no signs of rickets or skeletal dysplasia (Fig. 2). The infant's serum intact FGF23 levels dropped rapidly to 546 pg/mL (SI: 21.6 pmol/L) after 3 months and then to 151 pg/mL (SI: 6.0 pmol/L) after 7 months. In both mother and infant, genetic analysis identified a long interspersed nuclear element-1 retrotransposon insertion near exon 9 of the *PHEX* gene (Fig. 3).

**Table 1:** Longitudinal biochemical, treatment, and growth data of the infant with X-linked hypophosphatemia

Age	Day 2	Day 13	Day 30	3 months	7 months	8 months	12 months	15 months	20 months	Reference range
Treatment	None	Conventional	Conventional	Conventional	Conventional	Switch to burosumab (7 mg q2w)	Burosumab (10 mg q2w)	Burosumab (20 mg q2w)	Burosumab (20 mg q2w)	
Serum Phosphate*^a^*	4.2 mg/dL (1.36 mmol/L)	3.8 mg/dL (1.23 mmol/L)	4.4 mg/dL (1.42 mmol/L)	3.2 mg/dL (1.03 mmol/L)	3.9 mg/dL (1.26 mmol/L)	3.3 mg/dL (1.07 mmol/L)	3.9 mg/dL (1.26 mmol/L)	3.2 mg/dL (1.03 mmol/L)	3.6 mg/dL (1.16 mmol/L)	3.9-6.2 mg/dL (1 26-2.0 mmol/L) at 1 year
Serum calcium*^b^*	7.2 mg/dL (1.80 mmol/L)	9.9 mg/dL (2.48 mmol/L)	9.6 mg/dL (2.40 mmol/L)	9.8 mg/dL (2.45 mmol/L)	10.9 mg/dL (2.73 mmol/L)	10.3 mg/dL (2.58 mmol/L)	10.1 mg/dL (2.53 mmol/L)	10.4 mg/dL (2.60 mmol/L)	9.9 mg/dL (2.48 mmol/L)	8.8-10.6 mg/dL (2.20-2.64 mmol/L) at 1 year
ALP*^c^*	357 U/L (5.95 μkat/L)	510 U/L (8.50 μkat/L)	662 U/L (11.0 μkat/L)	751 U/L (12.5 μkat/L)	989 U/L (16.4 μkat/L)	760 U/L (12.7 μkat/L)	693 U/L (11.6 μkat/L)	675 U/L (11.3 μkat/L)	503 U/L (8.39 μkat/L)	138-469 U/L (2.30-7.82 μkat/L)at 1 year
Intact PTH	84 pg/mL (8.9 pmol/L)	—	—	96 pg/mL (10.2 pmol/L)	44 pg/mL (4.5 pmol/L)	—	—	—	—	10-65 pg/mL (1.1-6.9 pmol/L)
Intact FGF23	48 700 pg/mL (1933 pmol/L)	—	—	546 pg/mL (21.6 pmol/L)	151 pg/mL (6.0 pmol/L)	—	—	—	—	19.9-52.9 pg/mL (0.79-2.10 pmol/L)
TmP/GFR	3.34 mg/dL (1.08 mmol/L)	2.89 mg/dL (0.93 mmol/L)	3.21 mg/dL (1.04 mmol/L)	2.48 mg/dL (0.80 mmol/L)	3.47 mg/dL (1.12 mmol/L)	3.08 mg/dL (0.99 mmol/L)	3.52 mg/dL (1.14 mmol/L)	2.70 mg/dL (0.87 mmol/L)	3.15 mg/dL (1.01 mmol/L)	4.45-6.85 mg/dL (1.43-2.21 mmol/L) at 1 year
Urine Ca/Cr	0.01 mg/mg (0.003 mmol/mmol)	0.38 mg/mg (0.13 mmol/mmol)	0.19 mg/mg (0.06 mmol/mmol)	0.03 mg/mg (0.01 mmol/mmol)	0.1 mg/mg (0.03 mmol/mmol)	0.08 mg/mg (0.03 mmol/mmol)	0.25 mg/mg (0.08 mmol/mmol)	0.17 mg/mg (0.06 mmol/mmol)	0.24 mg/mg (0.08 mmol/mmol)	<0.3 mg/mg (<0.1 mmol/mmol)
Height	45.9 cm	47 cm	48 cm	54.5 cm	60.5 cm	65 cm	65.4 cm	70.9 cm	75.6 cm	
Weight	2.325 kg	2.530 kg	3.235 kg	5.615 kg	8.6 kg	8.7 kg	8.8 kg	8.8 kg	10.1 kg	

Values in parentheses are International System of Units (SI).

Conventional therapy was initiated on day 3 (alfacalcidol) and day 4 (phosphate). Burosumab was initiated at 8 months of age.

SI unit conversion factors: phosphate, mg/dL × 0.3229 = mmol/L; calcium, mg/dL × 0.25 = mmol/L; ALP, U/L × 16.67 × 10^−9^ = kat/L; intact PTH, pg/mL × 0.106 = pmol/L; intact FGF23, pg/mL × 0.0397 = pmol/L; TmP/GFR, mg/dL × 0.3229 = mmol/L; creatinine, mg/dL × 0.088 = mmol/L.

Abbreviations: ALP, alkaline phosphatase; Cr, creatinine; FGF23, fibroblast growth factor 23; PTH, parathyroid hormone; q2w, every 2 weeks; TmP/GFR, tubular maximum reabsorption of phosphate to glomerular filtration rate ratio.

^*a*^Reference range for serum phosphate differs according to age: newborn, 5.0 to 7.7 mg/dL (SI: 1.61-2.49 mmol/L); 1 month, 4.8 to 7.5 mg/dL (SI: 1.55-2.42 mmol/L); 3 months, 4.5 to 7.1 mg/dL (SI: 1.45-2.29 mmol/L); 6 months, 4.2 to 6.7 mg/dL (SI: 1.36-2.16 mmol/L); 12 months, 3.9 to 6.2 mg/dL (SI: 1.26-2.0 mmol/L); 24 months, 3.8 to 6.0 mg/dL (SI: 1.23-1.94 mmol/L).

^*b*^Reference range for serum calcium differs according to age: 0-12 months, 9.0 to 11.0 mg/dL (SI: 2.25-2.74 mmol/L): 12 months, 8.9 to 10.6 mg/dL (SI: 2.20-2.64 mmol/L); 24 months, 8.8 to 10.5 mg/dL (SI: 2.20-2.62 mmol/L).

^*c*^Reference range for serum ALP differs according to age: newborn, 186 to 564 U/L (SI: 3.10-9.40 μkat/L); 1 month, 179 to 567 U/L (SI: 2.98-9.45 μkat/L); 3 months, 168 to 567 U/L (SI: 2.80-9.45 μkat/L); 6 months, 147 to 553 U/L (SI: 2.45-9.22 μkat/L); 12 months, 138 to 469 U/L (SI: 2.30-7.82 μkat/L); 24 months, 144 to 438 U/L (SI: 2.40-7.30 μkat/L).

During the neonatal period, alfacalcidol (0.125 mcg/day) and oral phosphate (200 mg/day) were administered as conventional therapy. However, at 8 months of age, the patient had an elevated alkaline phosphatase (ALP) level of 760 U/mL (SI: 12.7 μkat/L) (reference range at 1 year, 138-469 U/mL [SI: 2.30-7.82 μkat/L]) and a low serum phosphate level of 3.3 mg/dL (SI: 1.07 mmol/L) (reference range at 1 year, 3.9-6.2 mg/dL [SI: 1.26-2.0 mmol/L]) despite receiving alfacalcidol (0.25 mcg/day) and oral phosphate (200 mg/day). At 8 months of age, the infant began receiving burosumab treatment (7 mg every 2 weeks) (Table 1). One year after burosumab initiation, the child's height was 75.6 cm and weight was 10.1 kg. Growth was monitored using the Japanese standard growth chart [7], and no adverse events associated with prenatal burosumab exposure during pregnancy or subsequent treatment were observed.

## Discussion

This case highlights the therapeutic dilemma of managing pregnancy in women receiving burosumab for XLH. Evidence for continued burosumab administration during pregnancy is extremely limited. While the current guidelines make a conditional recommendation for conventional therapy during pregnancy, they emphasize the importance of shared decision-making because of a lack of sufficient evidence. The patient's history of severe orthopedic complications as well as the risk of symptomatic rebound influenced our decision to continue burosumab treatment. Burosumab's efficacy in improving patient-reported outcomes such as pain and stiffness is well-established, and discontinuing it may severely compromise maternal caretaking capacity.

A striking finding was the neonate's transient, markedly elevated FGF23 level (48 700 pg/mL [SI: 1933 pmol/L]). Burosumab is an IgG1 antibody known to cross the placenta, particularly in the third trimester. We hypothesize that the extremely high FGF23 concentration is due to a combination of transplacental transfer of burosumab, which stabilizes circulating FGF23 by forming antibody-antigen complexes, and intrinsic FGF23 overproduction caused by the infant's inherited *PHEX* mutation. The mechanism for this elevation is likely the formation of burosumab-FGF23 immune complexes. Studies have demonstrated that burosumab binds to FGF23, preventing its degradation and clearance, resulting in an increase in total/intact FGF23 levels measured by immunoassays [8]. Furthermore, recent studies indicate that the presence of burosumab can interfere with certain commercially available FGF23 assays, resulting in markedly elevated values that do not necessarily reflect biologically active free FGF23 [9]. As a result, serial FGF23 measurements in this infant were not designed to provide a definitive assessment of disease activity. Rather, since burosumab was not administered after birth, we reasoned that the temporal decline in FGF23 levels might provide supportive information about the decreasing influence of maternally derived burosumab and help inform future treatment decisions.

The decrease of FGF23 to 151 pg/mL (SI: 6.0 pmol/L) over 7 months corresponds to the expected clearance of maternal IgG from the neonatal circulation. The persistence of elevated FGF23 at 7 months reflects the infant's intrinsic overproduction caused by an inherited *PHEX* mutation. Importantly, despite the *in utero* exposure and biochemical abnormalities, the infant did not experience adverse skeletal events, such as craniosynostosis, which has been reported in XLH [10]. The start of burosumab at 8 months was well-tolerated. Recent phase 3 trials and real-world data have demonstrated the safety and efficacy of burosumab in young children [11]. Although there were no obvious adverse skeletal findings observed during the first year of life, these findings should not be interpreted as proof of the efficacy of prenatal burosumab exposure. Interpretation of postnatal growth is limited as growth retardation in XLH is typically not visible during early infancy. Whether these findings apply to pregnancies in which the fetus does not inherit XLH is unclear. Therefore, the absence of short-term adverse outcomes does not guarantee safety, and careful neonatal monitoring is still necessary. Long-term follow-up is required to determine the significance of prenatal burosumab exposure.

## Learning points

- Burosumab effectively improves pain and mobility in adults with XLH, and its continued use during pregnancy may be considered when the risk of symptomatic rebound is high.
- Transplacental transfer of burosumab can result in markedly elevated serum FGF23 levels in the neonates, most likely due to antibody-antigen complex stability.
- In this case, the infant's FGF23 levels dropped significantly by 3 months of age but remained elevated due to inherited XLH; no adverse skeletal events or developmental delay were observed at 1 year after switching to burosumab therapy.

## Data Availability

Original data generated and analyzed during this study are included in this article.
